# Repurposing of Metformin to Improve Survival Outcomes in Patients With Upper Tract Urothelial Carcinoma

**DOI:** 10.1002/cam4.70567

**Published:** 2025-01-05

**Authors:** Hsiang Ying Lee, Po‐Hung Lin, See‐Tong Pang, Jen‐Kai Fang, Chung‐You Tsai, Yao‐Chou Tsai, Yung‐Tai Chen, Wei‐Chieh Chen, Hsin‐Chih Yeh, Wei‐Ming Li

**Affiliations:** ^1^ Department of Urology, Kaohsiung Medical University Hospital Kaohsiung Medical University Kaohsiung Taiwan; ^2^ Department of Urology, School of Medicine, College of Medicine Kaohsiung Medical University Kaohsiung Taiwan; ^3^ Graduate Institute of Clinical Medicine, College of Medicine Kaohsiung Medical University Kaohsiung Taiwan; ^4^ Division of Urology, Department of Surgery Chang Gung Memorial Hospital, Linkou Branch Taoyuan Taiwan; ^5^ School of Medicine, College of Medicine Chang Gung University Taoyuan Taiwan; ^6^ Graduate Institute of Clinical Medical Science, College of Medicine Chang Gung University Taoyuan Taiwan; ^7^ Division of Urology China Medical University Hospital Taichung Taiwan; ^8^ Department of Urology Far Eastern Memorial Hospital Taiwan; ^9^ Biomedical Informatics, Electrical and Communication Engineering College Yuan Ze University Taoyuan Taiwan; ^10^ Division of Urology, Taipei Tzu Chi Hospital Taiwan Urological Association Collaborative Research Organization Taipei Taiwan; ^11^ Department of Urology Taiwan Adventist Hospital Taipei Taiwan; ^12^ Department of Urology Postal Hospital Taipei Taiwan; ^13^ Department of Urology, National Taiwan University Hospital, College of Medicine National Taiwan University Taipei Taiwan; ^14^ Department of Urology Taipei Medical University Taipei Taiwan; ^15^ Department of Urology Kaohsiung Municipal Ta‐Tung Hospital Kaohsiung Taiwan; ^16^ Graduate Institute of Medicine, College of Medicine Kaohsiung Medical University Kaohsiung Taiwan; ^17^ Department of Urology Kaohsiung Medical University Gangshan Hospital Kaohsiung Taiwan; ^18^ Department of Urology Ministry of Health and Welfare Pingtung Hospital Pingtung Taiwan

**Keywords:** metformin, Survival outcome, upper tract urothelial carcinoma

## Abstract

**Purpose:**

Upper tract urothelial carcinoma (UTUC) presents a higher incidence rate in Taiwan compared to Western societies. The aim of this study is to investigate the potential of metformin in improving survival outcomes for patients with UTUC in Taiwan.

**Material and Methods:**

This retrospective study included 940 patients with UTUC and type 2 diabetes from the Taiwan UTUC Collaboration Group, spanning 21 hospitals from July 1988 to September 2023. Patients were divided into two groups: those treated with metformin (*n* = 215) and those without metformin treatment (*n* = 725). Parameters analyzed included age, BMI, renal function, tumor grade and location, and pathological staging. Oncological outcomes measured were overall survival (OS), cancer‐specific survival (CSS), and bladder recurrence‐free survival (BRFS). Statistical analysis involved the use of Student's *t*‐test, Mann–Whitney test, Chi‐squared test, Fisher's exact test, and Cox proportional hazard regression.

**Results:**

Significant differences were observed between the two groups in BMI, preoperative creatinine, eGFR, tumor location, tumor laterality, tumor size, and pathological grade and T stage. Patients treated with metformin exhibited a lower risk of CSS (HR = 0.619; *p* = 0.018) and improved OS (HR = 0.713; *p* = 0.024), although no significant association was found with BRFS (HR = 1.034; *p* = 0.791). The protective effect of metformin on OS was particularly significant in patients with advanced T stage, metastasis, and high‐grade tumors.

**Conclusion:**

The study suggests that metformin use in UTUC patients with diabetes is associated with improved OS and CSS but not BRFS. The underlying mechanisms warrant further investigation. Repurposing metformin, a well‐established and safe drug, may develop new therapeutic strategies for UTUC.

## 1 Introduction

Upper tract urothelial carcinoma (UTUC) has higher incidence rate in Taiwan compared to the western society. It accounts for approximately 40% of urothelial carcinoma (UC) with a higher incidence in females than males [1, 2]. UTUC is characterized by relatively aggressive biological behavior and poorer differentiation compared to bladder cancer. Therefore, it necessitates a precise evaluation of disease progression and tumor invasiveness in each case [3]. Factors such as age, tumor grade, stage, sessile tumor growth, lymphovascular invasion (LVI), lymph node involvement, necrosis, and tumor location have been identified in the literature as associated with the prognosis of UTUC patients [4]. Previous studies demonstrated that smoking, environmental factors including exposure to aristolochic acid in Chinese herbal products and arsenic contamination are related to risk of UTUC. Due to the advanced characteristics of UTUC, it is important to identify factors or intervention that can reduce the progression of cancer.

Metformin is the most widely used oral medication to treat type 2 diabetes mellitus (DM). From previous studies, metformin has potential role to improve survival outcomes in various cancer including urologic cancers [5]. In particular, the anticancer characteristics of metformin have been studied in lung cancer, breast cancer, pancreatic cancer and colon cancer, prostate cancer, bladder cancer [6, 7]. The possible antitumor mechanism may come from direct impact on cancer cells through activation of AMPK pathway and inhibition of the regulatory associated protein of mTORC1. In addition, metformin also present effects on tumor microenvironment, tumor angiogenesis and related to inflammatory factors and immune activation [8].

Because of the extended research and development period for new drugs, there is a growing interest in exploring the use of established and safe drugs already on the market to advance tumor treatment. As such, researchers are investigating the potential of metformin in treating various cancer. However, findings regarding metformin's protective effects in UTUC is lack. Based on the high incidence rate of UTUC in Taiwan, we conducted this research to verify if metformin has similar effect with other cancers.

## 2 Material and Methods

### 2.1 Patient Collection

We conducted this retrospective study from the Taiwan UTUC Collaboration Group includes 21 participating hospitals. This research was approved by the Institutional Review Board (KMUHIRB‐E(I)‐20180214). A total of 6208 UTUC patients were included from July 1988 to September 2023. After excluding patients without receiving surgery, without receiving neoadjuvant and adjuvant systemic therapy or loss follow up, or incomplete data and patients without DM comorbidity, we finally included 940 patients. There are 725 DM patients without metformin treatment and 215 DM patients using metformin for controlling sugar. The duration of metformin exposure is at least over 6 months.

In addition to different anti‐diabetic drug interventions, we collected various parameters for analysis, including age, body mass index (BMI), renal function, gender, tumor grade, tumor location, tumor focality, pathological T and N stage who received lymph node dissection, tumor laterality, tumor size, concomitant carcinoma in situ (CIS), lymphovascular invasion (LVI).

### 2.2 Definitions and Endpoints

The samples obtained from radical surgery were assessed by pathologists using identical criteria. Pathological staging from the 2010 TNM (tumor, lymph node, metastasis) system, and tumor grading adhered to the 2004 World Health Organization/International Society of Urologic Pathology consensus classification. The regular follow‐up program strictly adhered to standard guidelines. The study aimed to compare oncological outcomes between UTUC patients with DM receiving metformin treatment or not. Overall survival (OS), cancer‐specific survival (CSS), and bladder recurrence‐free survival (BRFS) were analyzed. The cause of death was determined by the attending physician or death certificate.

### 2.3 Statistical Analysis

Student's *t*‐test and Mann–Whitney test were used for continuous variables that were normally distributed or not, respectively. Continued variables were reported as mean (SD) or median (Q1–Q3) and frequencies (%). We used the Chi‐squared test and Fisher's exact test for categorical variables. The hazard ratio (HR) with its 95% confidence interval (CI) showed the association between each predictor variable and OS, CSS, BRFS. It was calculated with Cox proportional hazard regression. Variables with a *p*‐value < 0.05 were selected into the adjusted model by stepwise selection in univariate Cox regression. The statistical analysis was performed using SAS software (version 9.4, SAS Institute Inc., Cary, NC), and statistical significance was assigned as *p* < 0.05.

## 3 Results

Nine Hundred and Forty patients were included and divided into groups with metformin (*n* = 215) and without metformin (*n* = 725). The demographic and pathological characteristics of UTUC patients in the groups with or without metformin were compared in Table 1. There were differences in BMI (*p* = 0.024), preoperative creatinine (*p* < 0.001), preoperative eGFR (*p* < 0.001), tumor location (*p* = 0.004), tumor laterality (*p* = 0.014), tumor size (*p* < 0.001) and tumor pathological grade (*p* = 0.016) and T stage (*p* = 0.026) between the two groups.

**TABLE 1:** Comparison of clinicopathological data between DM without metformin and with metformin in UTUC patients.

Variables	Without metformin (*n* = 725)	With metformin (*n* = 215)	*p*
Age (years)	71.38 (65.28–77.82)	70.47 (64.32–77.26)	0.422
BMI (kg/m^2^)	24.93 (22.6–27.14)	25.72 (23.44–28.03)	0.024*
eGFR (mL/min/1.73 m^2^) before surgery	39.95 (21.17–58.33)	56.38 (41.39–74.39)	< 0.001*
Gender			0.058
Male	308 (77.78)	88 (22.22)	
Female	417 (76.65)	127 (23.35)	
Grade			0.016*
Low grade	86 (70.49)	36 (29.51)	
High grade	632 (78.22)	176 (21.78)	
Location			0.004*
Renal pelvis	284 (76.34)	88 (23.66)	
Ureter	290 (76.92)	87 (23.08)	
Synchronous	149 (78.84)	40 (21.16)	
Multifocality			0.059
No	449 (77.55)	130 (22.45)	
Yes	265 (76.37)	82 (23.63)	
Pathological N stage			0.214
pN0/Nx	14 (82.35)	3 (17.65)	
pN1/N2	704 (76.86)	212 (23.14)	
Pathological T stage			0.026*
pT < 2	348 (75.16)	115 (24.84)	
pT ≥ 2	371 (78.77)	100 (21.23)	
Laterality			0.014*
Left	375 (77.64)	108 (22.36)	
Right	341 (76.29)	106 (23.71)	
Both	8 (88.89)	1 (11.11)	
Tumor size			< 0.001*
< 1 cm	35 (68.63)	16 (31.37)	
< 2 cm	130 (71.82)	51 (28.18)	
< 3 cm	111 (69.81)	48 (30.19)	
≥ 3 cm	282 (74.02)	99 (25.98)	
Carcinoma in situ			0.058
No	554 (76.41)	171 (23.59)	
Yes	164 (78.85)	44 (21.15)	
Lymphovascular invasion			0.079
No	587 (77.03)	175 (22.97)	
Yes	128 (78.05)	36 (21.95)	
Metastasis			0.007*
No	543 (79.04)	144 (20.96)	
Yes	102 (70.34)	43 (29.66)	

Abbreviation: BMI, body mass index.

* *p* < 0.05.

### 3.1 Oncological Outcomes

**FIGURE 1:**
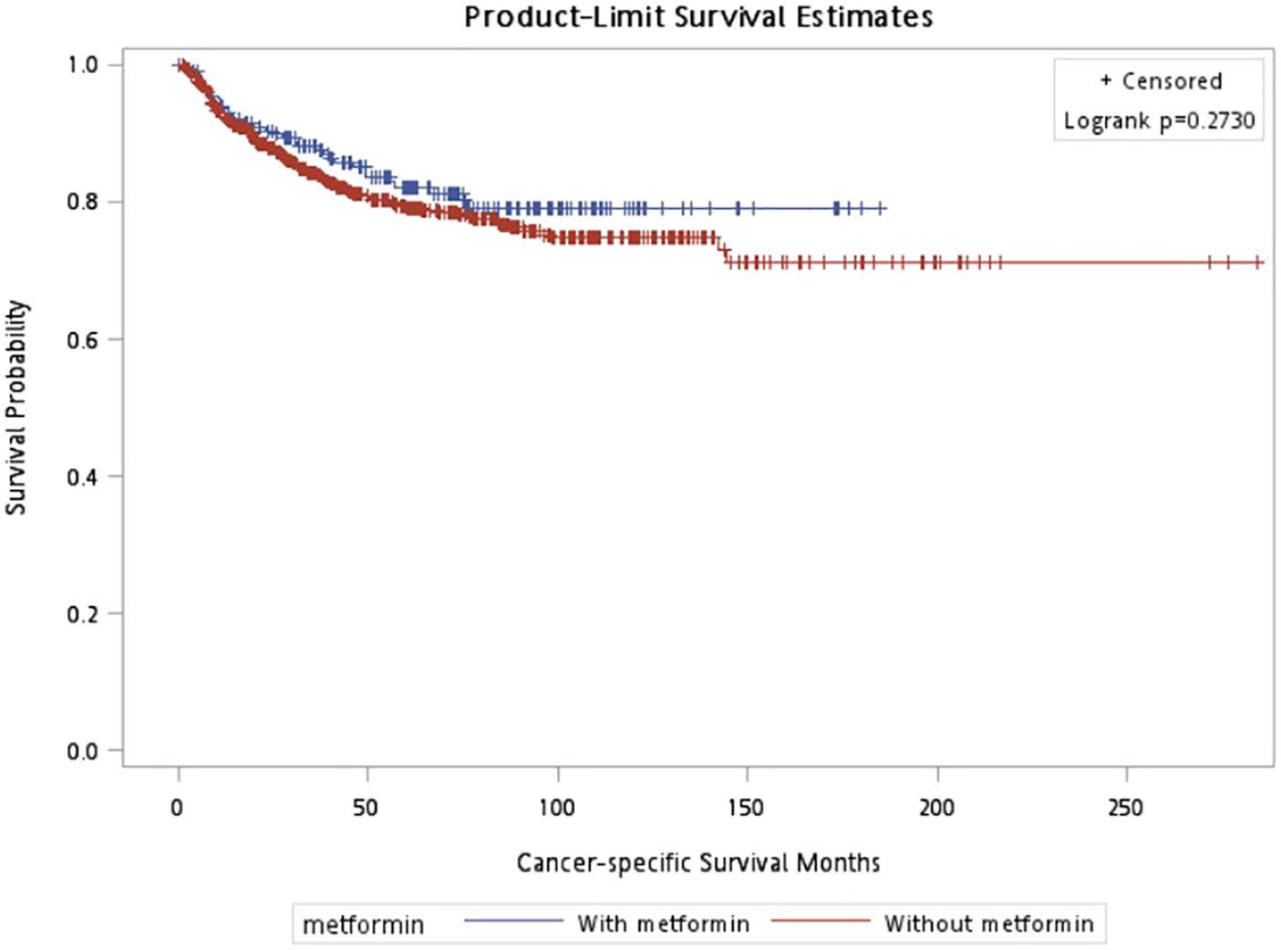
Cancer‐specific survival, *p* = 0.273.

**FIGURE 2:**
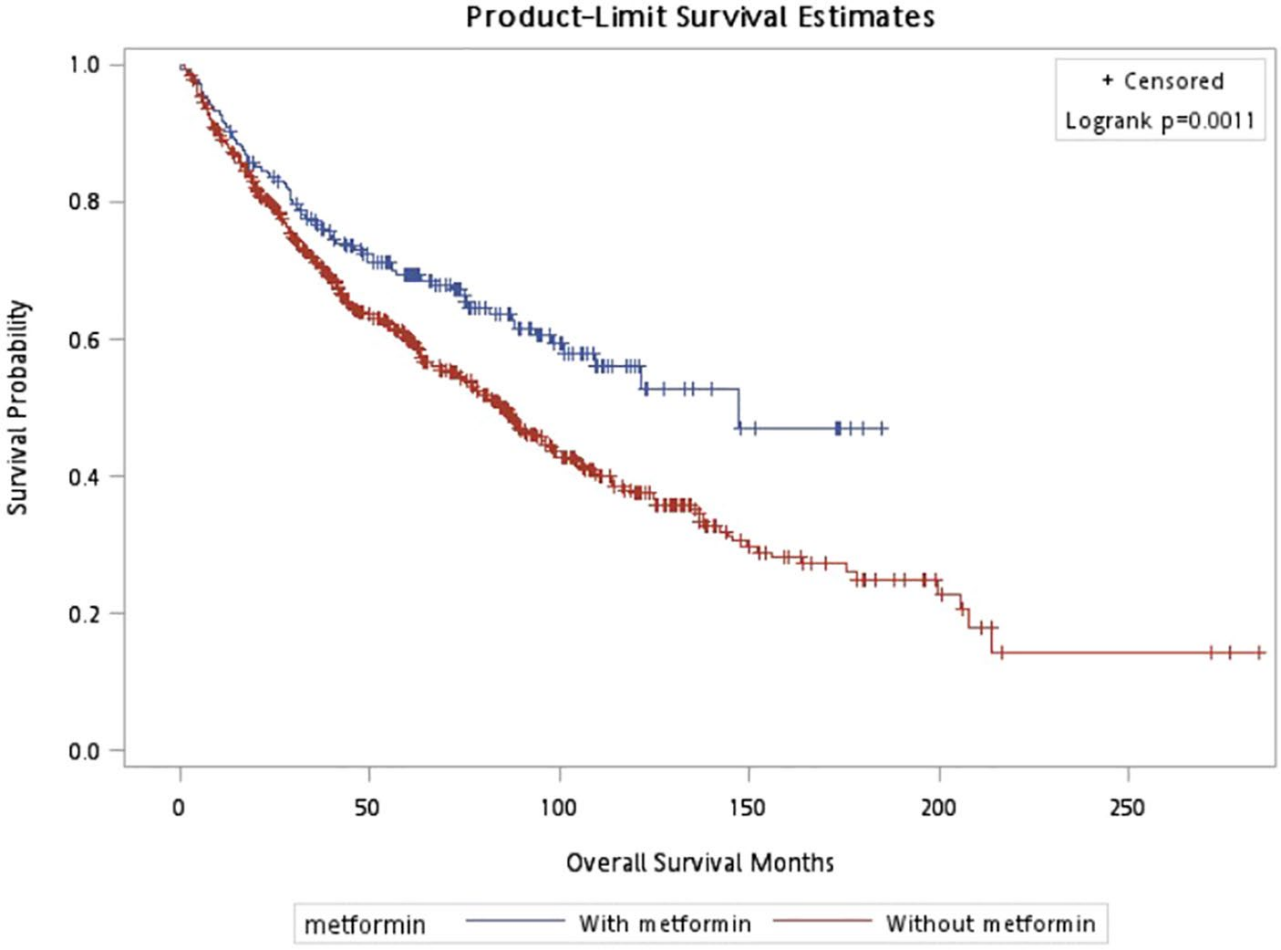
Overall survival, *p* = 0.001.

**FIGURE 3:**
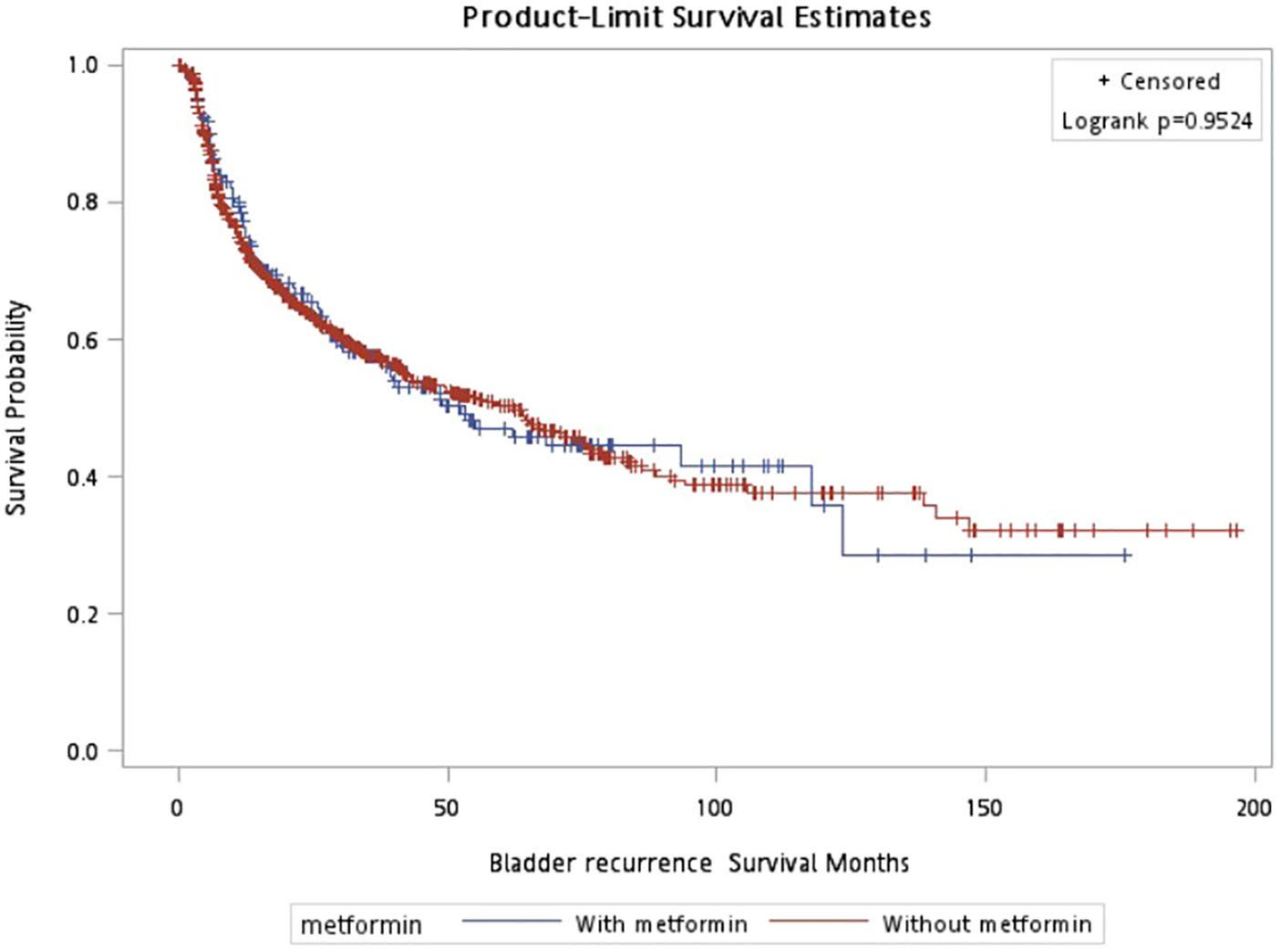
Bladder recurrence‐free survival, *p* = 0.952.

In CSS, pathologic T stage, metastasis, age, and LVI were included in the adjusted model. Patients with metformin had a lower risk of cancer related death (HR = 0.619; 95% CI (0.417–0.920), *p* = 0.018) than those without using metformin (Table 2). The survival curve in CSS showed no significant difference (*p* = 0.273) (Figure 1). Older patients, tumor with LVI, metastasis during follow up, advanced T stage have worse CSS. As for OS, the stepwise method selected age, BMI, metastasis, pathologic T stage, and LVI into the adjusted model. The significant protective effect of metformin on OS (HR = 0.713; 95% CI (0.532–0.956), *p* = 0.024) was observed (Table 3) The survival curve in OS also showed better survival outcome in patients with metformin usage (*p* = 0.001) (Figure 2). In addition, older patients, patients with low BMI, tumor with LVI, metastasis, advanced T stage have lower OS. In Table 4 and Figure 3, it showed no significant association if patients under metformin usage or not with BRFS (HR = 1.034; 95% CI (0.808–1.322), *p* = 0.791).

**TABLE 2:** Cancer specific‐survival analysis of UTUC with DM patients.

	Crude Cox model		Adj Cox model	
Variables	cHR (95% CI)	*p*	aHR (95% CI)	*p*
Age	1.023 (1.005–1.042)	0.013*	1.023 (1.004–1.043)	0.020*
BMI	0.966 (0.919–1.015)	0.167		
CIS				
No	1			
Yes	0.924 (0.626–1.363)	0.689		
Grade				
Low grade	1			
High grade	2.952 (1.506–5.788)	0.002*		
Location				
Renal pelvis	1			
Ureter	1.135 (0.788–1.634)	0.497		
Synchronous	1.741 (1.161–2.610)	0.007*		
LVI				
No	1		1	
Yes	3.389 (2.421–4.744)	< 0.001*	1.853 (1.290–2.660)	0.001*
Metastasis				
No	1		1	
Yes	22.123 (15.202–32.195)	< 0.001*	17.236 (11.515–25.801)	< 0.001*
Metformin				
Without metformin	1		1	
With metformin	0.811 (0.557–1.181)	0.274	0.619 (0.417–0.920)	0.018*
Multifocality				
No	1			
Yes	1.72 (1.253–2.361)	< 0.001*		
Pathological N stage				
pN0/Nx	1			
pN1/N2	9.283 (4.859–17.735)	< 0.001*		
eGFR (mL/min/1.73 m^2^) before surgery	0.993 (0.987–0.999)	0.035*		
Pathological T stage				
pT < 2	1		1	
pT ≥ 2	6.248 (4.122–9.473)	< 0.001*	2.324 (1.468–3.678)	< 0.001*
Gender				
Male	1			
Female	0.738 (0.539–1.009)	0.057		
Laterality				
Left	1			
Right	1.060 (0.775–1.450)	0.715		
Both	—	—		
Size				
< 1 cm	1			
< 2 cm	1.057 (0.392–2.847)	0.913		
< 3 cm	1.377 (0.517–3.670)	0.522		
≥ 3 cm	2.786 (1.130–6.869)	0.026*		

Abbreviations: BMI, body mass index; CI, confidence interval; CIS, carcinoma in situ; HR, hazard ratio; LVI, lymphovascular invasion.

* *p* < 0.05.

**TABLE 3:** Overall survival analysis of UTUC with DM patients.

	Crude Cox model		Adj Cox model	
Variables	cHR (95% CI)	*p*	aHR (95% CI)	*p*
Age	1.045 (1.033–1.058)	< 0.001*	1.045 (1.029–1.061)	< 0.001*
BMI	0.940 (0.911–0.970)	< 0.001*	0.954 (0.920–0.989)	0.010*
CIS				
No	1			
Yes	0.977 (0.771–1.238)	0.848		
Grade				
Low grade	1			
High grade	1.605 (1.169–2.201)	0.003*		
Location				
Renal pelvis	1			
Ureter	1.329 (1.067–1.656)	0.011*		
Synchronous	1.694 (1.305–2.200)	< 0.001*		
LVI				
No	1		1	
Yes	2.314 (1.846–2.902)	< 0.001*	1.878 (1.414–2.494)	< 0.001*
Metastasis				
No	1		1	
Yes	4.919 (3.916–6.178)	< 0.001*	4.604 (3.473–6.104)	< 0.001*
Metformin				
Without metformin	1		1	
With metformin	0.663 (0.517–0.850)	0.001*	0.713 (0.532–0.956)	0.024*
Mutifocality				
No	1			
Yes	1.459 (1.199–1.775)	< 0.001*		
Pathological N stage				
pN0/Nx	1			
pN1/N2	5.490 (3.075–9.802)	< 0.001*		
eGFR (mL/min/1.73 m^2^) before surgery	0.988 (0.984–0.992)	< 0.001*		
Pathological T stage				
pT < 2	1		1	
pT ≥ 2	2.117 (1.737–2.580)	< 0.001*	1.489 (1.124–1.972)	0.006*
Gender				
Male	1			
Female	0.970 (0.798–1.179)	0.760		
Laterality				
Left	1			
Right	1.009 (0.832–1.224)	0.925		
Both	1.660 (0.684–4.029)	0.263		
Size				
< 1 cm	1			
< 2 cm	1.442 (0.827–2.514)	0.197		
< 3 cm	1.473 (0.838–2.590)	0.178		
≥ 3 cm	2.070 (1.222–3.507)	0.007*		

Abbreviations: BMI, body mass index; CI, confidence interval; CIS, carcinoma in situ; HR, hazard ratio; LVI, lymphovascular invasion.

* *p* < 0.05.

**TABLE 4:** Bladder recurrence‐free survival analysis of UTUC with DM patients.

Variables	Crude Cox model		Adj Cox model	
	cHR (95% CI)	*p*	aHR (95% CI)	*p*
Age	1.029 (1.017–1.041)	< 0.001*	1.027 (1.015–1.040)	< 0.001*
BMI	0.974 (0.947–1.002)	0.072		
CIS				
No	1			
Yes	1.202 (0.959–1.506)	0.110		
Grade				
Low grade	1			
High grade	1.272 (0.945–1.711)	0.112		
Location				
Renal pelvis	1		1	
Ureter	1.201 (0.959–1.503)	0.110	1.238 (0.975–1.572)	0.080
Synchronous	1.615 (1.246–2.092)	< 0.001*	1.473 (1.117–1.942)	0.006*
LVI				
No	1			
Yes	1.689 (1.329–2.145)	< 0.001*		
Metastasis				
No	1		1	
Yes	2.009 (1.557–2.591)	< 0.001*	1.645 (1.254–2.158)	< 0.001*
Metformin				
Without metformin	1		1	
With metformin	0.993 (0.79–1.248)	0.953	1.034 (0.808–1.322)	0.791
Mutifocality				
No	1			
Yes	1.409 (1.154–1.72)	< 0.001*		
Pathological N stage				
pN0/Nx	1			
pN1/N2	2.152 (1.112–4.167)	0.023*		
eGFR (mL/min/1.73 m^2^) before surgery	0.995 (0.991–0.999)	0.009*		
Pathological T stage				
pT < 2	1		1	
pT ≥ 2	1.724 (1.413–2.102)	< 0.001*	1.429 (1.146–1.782)	0.002*
Gender				
Male	1		1	
Female	0.645 (0.53–0.784)	< 0.001*	0.639 (0.516–0.791)	< 0.001*
Laterality				
Left	1			
Right	1.005 (0.826–1.223)	0.960		
Both	1.125 (0.360–3.519)	0.839		
Size				
< 1 cm	1			
< 2 cm	1.057 (0.643–1.738)	0.826		
< 3 cm	1.419 (0.868–2.318)	0.163		
≥ 3 cm	1.500 (0.944–2.383)	0.086		

Abbreviations: BMI, body mass index; CI, confidence interval; CIS, carcinoma in situ; HR, hazard ratio; LVI, lymphovascular invasion.

* *p* < 0.05.

In the stratified analysis, the protective effect of metformin on OS showed significant in patients with any pathological T stage, metastasis during follow up, tumors with LVI or not, high grade tumor, excluding low grade tumor. The relationship between metformin usage and CSS was observed in those with high grade tumor and metastasis. The significance association between metformin usage and BRFS was not seen in stratifications (Table 5).

**TABLE 5:** Stratified survival analysis of UTUC with DM patients.

Stratification	OS		CSS		BRFS	
	aHR (95% CI)	*p*	aHR (95% CI)	*p*	aHR (95% CI)	*p*
Pathological T stage						
≥ T2	0.698 (0.500–0.975)	0.035*	0.676 (0.439–1.040)	0.075	1.158 (0.835–1.607)	0.379
< T2	0.449 (0.278–0.722)	0.001*	0.432 (0.152–1.227)	0.115	0.772 (0.524–1.138)	0.192
Metastasis						
No	0.630 (0.444–0.894)	0.010*	0.813 (0.356–1.852)	0.621	1.080 (0.814–1.433)	0.595
Yes	0.537 (0.348–0.827)	0.005*	0.578 (0.368–0.907)	0.017*	0.756 (0.456–1.254)	0.279
Grade						
High grade	0.566 (0.421–0.760)	< 0.001*	0.608 (0.404–0.913)	0.017*	0.959 (0.730–1.26)	0.765
Low grade	0.693 (0.306–1.568)	0.378	1.525 (0.094–24.618)	0.766	1.192 (0.615–2.311)	0.603
LVI						
LVI	0.483 (0.281–0.829)	0.008*	0.555 (0.280–1.101)	0.092	0.728 (0.419–1.265)	0.260
Non‐LVI	0.677 (0.495–0.926)	0.015*	0.695 (0.429–1.127)	0.140	1.056 (0.799–1.396)	0.702

*Note:* Compare Kaplan–Meier curves between patients with and without metformin by log‐rank test. Adjusted age, Metastasis, pT, and LVI.

Abbreviations: BRFS, bladder recurrence‐free survival; CI, confidence interval; CSS, cancer‐specific survival; HR, hazard ratio; LVI, Lymphovascular invasion; OS, overall survival.

* *p* < 0.05.

## 4 Discussion

Metformin is a safe and effective oral antidiabetic drug in glycemic control which emerging as a promising candidate for the prevention and treatment of malignant tumors. Recent years have seen encouraging results from the use of metformin in managing various types of cancer. However, clinical outcomes have varied [9, 10, 11]. Our database showed that metformin usage might be associated with a significant improvement in the OS and CSS of UTUC.

UTUC in Taiwan exhibits distinct characteristics and high incidence rate compared to Western countries, which may be attributed to genetic, environmental, and dietary factors specific to the region [12, 13]. Chang YH et al. indicated that there was a high occurrence in women, particularly in renal pelvis cancer between 1985 and 2019 [2]. Additionally, it showed increasing trend in the incidence of renal pelvis cancer in women from 1985 to 1999. Furthermore, UTUC in Taiwanese patients often presents with aggressive behavior and a high rate of recurrence. Chen CH et al. demonstrated that people living in Taiwan may exposure both arsenic and aristolochic acid carcinogens, the risk of developing UTUC may additive [14]. Improving long‐term survival rates and quality of life for UTUC patients remains a significant challenge.

The anti‐tumor effects of metformin have attracted increasing attention recently. Previous research highlighted the mechanism involving the activation of tumor cell cycle and associated signaling pathways [15]. These encompass the stimulation of AMPK‐associated pathways, the enhancement of apoptosis in cancer cells, and the suppression of mitochondrial metabolism [16, 17, 18]. Such studies have illuminated the crucial function of metformin in cancer treatment and the pathways that are regulated. The insights gained from these studies offer considerable potential for improving clinical practices and drug development in cancer therapy. Chen et al. [19] utilized MR analysis to reveal the genetic connections between metformin use and the risk of prevalent cancers. Shen Z. et al. [20] conducted bladder cancer cell line experiments to explore the possible molecular mechanisms of metformin. They discovered metformin can inhibit cancer cell migration and proliferation in terms of inhibiting bladder cancer progression.

Some previous studies have revealed that the PI3K/AKT/mTOR was dysregulated in over 40% of patients with urothelial carcinoma [21]. This finding can be used to infer that metformin can inhibit the growth of bladder cancer cell through the PI3K pathway which was identified with in vitro study [20]. Furthermore, combination therapy may also have additive effect on survival of cancer. Almaimani RA et al. demonstrated that combinations of metformin with 5‐fluorouracil or even triple therapy regimens effectively induced anticancer activity, which included cell cycle arrest and apoptosis, compared to single drug treatments. This enhanced anticancer effect is likely due to the attenuation of the PI3K/AKT/mTOR oncogenic pathway [22].

Our current study unveiled the potential role of metformin in improving OS and CSS of UTUC. From previous systematic review, the use of metformin may be related to improvement in recurrence, CSS and OS of prostate cancer and the progression of kidney cancer [5]. In prostate cancer, the beneficial impact especially on patients who received radical radiotherapy. This effect may be due to the involvement of the AMPK pathway in managing how cells react to radiation treatment [23]. On the contrary, several research did not show significant positive correlation between metformin and survival outcomes [24, 25]. However, the meta‐analysis of Liu et al. demonstrated that metformin emerged as a substantial protective factor in reducing the risk of bladder cancer instead of improving survival outcomes after including six studies altogether for analysis [7]. Despite this, Malte Rieken et al. have demonstrated that diabetic patients who do not use metformin seem to face a higher risk of CSS and OS compared to patients without DM [26]. Regarding the impact of metformin on UTUC, previous study has also found positive effects. M. Rieken et al. [27] found DM patients with UTUC who did not use metformin had a worse CSS and higher disease recurrence rate. The definition of recurrence is tumor progression in operative field, regional lymph nodes and/or distant metastasis.

There are some limitations in our study. First, it is a retrospective design research, however it is the largest collaboration study of UTUC. Second, the timing of metformin exposure, whether before or after the diagnosis of UTUC is unknown. Further prospective study warrants to clarify if the timing of metformin usage can influence the prognosis of UTUC. Third, we must clarify whether the severity of DM in this group of patients or the use of metformin itself affects the outcomes of UTUC.

## 5 Conclusions

UTUC patients with DM who use metformin appear to be lower risk of CSS and OS but not bladder recurrence than patients without usage of metformin in our study. However, the underlying mechanisms and possible effects of metformin on UTUC require further clarification. Nevertheless, repurposing old drugs is a potential candidate for development of new therapeutic role for UTUC.

## Author Contributions

**Hsiang Ying Lee:** data curation (lead), writing – original draft (lead). **Po‐Hung Lin:** resources (equal), supervision (equal). **See‐Tong Pang:** resources (equal), supervision (equal). **Jen‐Kai Fang:** resources (equal), supervision (equal). **Chung‐You Tsai:** resources (equal), supervision (equal). **Yao‐Chou Tsai:** conceptualization (equal), resources (equal), supervision (equal). **Yung‐Tai Chen:** resources (equal), supervision (equal). **Wei‐Chieh Chen:** resources (equal), supervision (equal). **Hsin‐Chih Yeh:** supervision (equal), writing – review and editing (equal). **Wei‐Ming Li:** conceptualization (equal), supervision (equal), writing – review and editing (equal).

## Consent

A waiver was granted by the IRB/Ethics Committee for this purpose. This research was approved by the Institutional Review Board (KMUHIRB‐E(I)‐20180214).

## Conflicts of Interest

The authors declare no conflicts of interest.

## Data Availability

The authors confirms that all data generated or analyzed during this study are included in this published article.
